# Air pollution across One Health domains 

**DOI:** 10.2471/BLT.26.295988

**Published:** 2026-07-27

**Authors:** David Rojas-Rueda

**Affiliations:** a Department of Environmental and Radiological Health Sciences, Colorado State University, Environmental Health Building, 1681 Campus Delivery, Fort Collins, Colorado 80523-1681, United States of America.

In June 2023, smoke from Canadian wildfires settled over much of North America for weeks. Emergency departments from Montreal, Canada to New York, United States were filled with people in respiratory distress.^1^ In Colorado, United States, dairy operations reported drops in milk production.^2^ Field biologists later recorded depressed growth in wild bird nestlings exposed to only a few days of elevated particulate matter.^3^ One exposure reached human, animal and ecosystem health at the same time, across a continent, and no single agency, surveillance system or assessment tool was built to capture the full event.

This gap is institutional. In 2022, the Quadripartite organizations, the World Health Organization (WHO), Food and Agriculture Organization of the United Nations, World Organisation for Animal Health and United Nations Environment Programme, launched the *One Health joint plan of action (2022–2026): working together for the health of humans, animals, plants and the environment*, organized around six action tracks.^4^ Air pollution appears in the plan once, in a background paragraph noting that pollution from fossil fuels and other sources harms human and animal health, biodiversity and water quality.^4^ None of the six tracks provide any operational elements for air pollution: there are no activities, deliverables or indicators. However, air pollution is a test of whether One Health reaches beyond its origins in infectious disease to the wider environmental determinants of health. The One Health definition adopted in 2021 by the One Health High-Level Expert Panel explicitly encompasses environmental determinants beyond zoonotic threats, making this omission a gap in implementation rather than in scope. As the current plan reaches the end of its cycle, the omission is worth correcting because the evidence that air pollution is a One Health problem is now substantial.

## Evidence across the three domains

The *State of global air 2025* report attributes 7.9 million deaths in 2023 to ambient and household air pollution, ranking it second only to high blood pressure among risk factors for human death.^5^ Of these deaths, an estimated 90% fall in low- and middle-income countries. Roughly one third of the global population breathes air that exceeds the WHO interim target of 35 µg/m^3^ for annual mean fine particulate matter.^5^ The WHO 2021 air quality guidelines^6^ lowered recommended targets in response to evidence of harm well below levels once considered safe, yet the gap between guideline values and ambient concentrations has widened rather than narrowed in much of the world.^5^ Although strong evidence from decades of research shows the magnitude of the risk, action is still insufficient. National air quality monitoring is still uneven, particularly in low- and middle-income countries,^5^ and many countries lack the capacity to run health impact assessments. In addition, the tools that do exist stop at human health, which is the only One Health domain with established cohorts, burden estimates and assessment tools. Comparable tools and routine burden estimates for air pollution do not yet exist for either animal health or ecosystem health. 

Evidence from animal health has accumulated faster than it has been translated into policy. For example, a study linked 87 108 dairy cow deaths in Belgium to ambient pollution, finding that a 10 µg per cubic metre rise in same-day nitrogen dioxide raised mortality by an estimated 9%.^7^ Dairy cattle make unusually consistent study populations: genetically similar, registered at birth and death, and free of the smoking and occupational exposures that complicate human studies. A recent review synthesized the growing literature on wildfire smoke and dairy cattle, reporting reduced milk production and measurable health effects.^2^ Air pollution also affects companion animals: a study drawing on five years of veterinary records in the United Kingdom of Great Britain and Northern Ireland found that higher fine particulate levels raised veterinary visits for both cats and dogs, and estimated that meeting the WHO guideline would prevent around 80 000 visits a year in the United Kingdom alone.^8^ In wildlife, a review of 41 studies spanning multiple continents documented respiratory distress, immune suppression and altered behaviour following wildfire smoke.^3^ A 2024 meta-analysis of 120 studies found that air pollution, ozone most of all, reduces the performance of beneficial invertebrates including pollinators, at concentrations below current air quality standards.^9^ The effects in animals mirror those in humans: respiratory and cardiovascular stress, immune suppression, reduced reproduction and premature death. The difference is that almost none of these effects in animals are captured in any assessment or policy framework.

Evidence also shows that the ecosystem domain is where air pollution effects loop back to human and animal health. Vegetation in the United States removes millions of tonnes of pollutants each year, a function that a study has valued at billions of United States dollars, yet the same pollutants damage the vegetation doing the filtering, so capacity falls as exposure rises.^10^ Ozone suppresses crop yields; another study projected that it will compromise efforts to raise global wheat production, with direct consequences for food security.^11^ Recent analysis warns that ground-level ozone may be approaching concentrations that threaten bird populations.^12^ However, the relationship is also bidirectional, as intensive livestock production emits ammonia that forms secondary particulate matter. One study tied food production in the United States to roughly 15 900 human deaths a year from fine particulate matter. Approximately 80% of those deaths are linked to animal-based foods, primarily through ammonia emissions.^13^ The 2023 wildfire season showed all these effects during one event, with concurrent human respiratory crises, dairy losses, depressed bird growth and vegetation damage.

## The next One Health action plan

The current Quadripartite Joint Plan of Action, which shapes national plans worldwide, should be the starting point of the next One Health action plan. Four additions would move air pollution from background mention to operational commitment: (i) make air pollution a priority; (ii) commit to cross-sectoral surveillance; (iii) set up a roadmap for assessments beyond human endpoints; (iv) establish shared ministerial accountability. None of these additions requires building from scratch. Because the plan cascades into national action plans, each addition also has a counterpart that national and subnational authorities can act on directly (Table 1).

**Table 1:** Proposed additions to One Health action plans for air pollution, with corresponding actions for national and subnational authorities

Element	What One Health action plans should include	What national and subnational authorities can do now	Example indicator
Named environmental priority	List air pollution as an explicit item with a deliverable and an indicator	Name air pollution and designate a lead agency in the national One Health action plan	Air pollution named; lead agency designated
Cross-sectoral surveillance	Commit to linking human health, veterinary and ecosystem monitoring data within shared airsheds	Link local air quality monitoring to veterinary registries and ecological monitoring networks	Number of shared-airshed surveillance pilots established
Assessment beyond human endpoints	Commission a roadmap to extend health impact assessment tools (AirQ+, BenMAP-CE, HRAPIE) to animal and ecosystem outcomes	Add livestock, crop, pollinator and veterinary costs to local cost-benefit analyses of air quality measures	Share of national assessments including non-human endpoints
Shared ministerial accountability	Require joint air quality targets across health, environment, agriculture and plant-health ministries	Set joint targets across the corresponding national ministries	Joint targets adopted across ministries (yes or no)

BenMAP-CE: Environmental Benefits Mapping and Analysis Program, community edition; HRAPIE: Health risks of air pollution in Europe.

The next One Health action plan should give air pollution a named place under the environmental track, with at least one deliverable and one indicator rather than a single descriptive sentence. An indicator as basic as the share of Member States that link air quality data to animal or ecosystem health monitoring would make progress visible and comparable.

The plan should commit to cross-sectoral surveillance that connects systems already running. Public health surveillance, veterinary registries and ecological monitoring are rarely engaged with one another, yet the Belgian dairy work shows what linkage reveals: a dose–response relationship that neither the health system nor the veterinary system could detect alone.^7^ Because monitoring networks were built around human exposure, they also leave remote areas, including forests, poorly covered, so any effort will need to extend observation beyond the population centres where air quality monitoring stations cluster. Piloting shared airshed surveillance and then scaling it would build on existing infrastructure rather than duplicating it.

The plan should also set a roadmap for assessment tools that currently stop at human health. Extending health impact assessment tools and health risk assessments, such as WHO AirQ+, Environmental Benefits Mapping and Analysis Program, community edition and the Health Risks of Air Pollution in Europe, to animal and ecosystem outcomes would be a necessary advance. While the extension is a medium-term task, the Quadripartite can commission the roadmap now, and in the meantime ask that cost-benefit analyses of air quality measures acknowledge livestock, crop, pollinator and veterinary costs alongside human ones. Including those dimensions rarely changes which policies pass a cost-benefit test, but it often changes substantially the size of the estimated benefit.

The plan should also assign accountability across relevant ministries. Air quality targets written into national One Health action plans and shared across environment, health, agriculture and plant-health authorities, turn a cross-domain threat into a cross-domain mandate.

The Quadripartite Joint Plan of Action describes itself as a living document open to adjustment, so none of these actions have to wait for the next planning cycle to begin.^4^ The One Health High-Level Expert Panel that advises the Quadripartite is well placed to champion the integration of air pollution into the current plan. The evidence and many of the systems for assessing it already exist, but air pollution is yet to be treated as a One Health problem.
